# Scan Path Optimization and YOLO-Based Detection for Defect Inspection of Curved and Glossy Surfaces

**DOI:** 10.3390/s26103026

**Published:** 2026-05-11

**Authors:** Min-Gyu Kim, Chibuzo Nwabufo Okwuosa, Jang-Wook Hur

**Affiliations:** Department of Mechanical Engineering (Department of Aeronautics, Mechanical and Electronic Convergence Engineering), Kumoh National Institute of Technology, 61 Daehak-ro, Gumi-si 39177, Gyeonsangbuk-do, Republic of Korea; 2025210408@kumoh.ac.kr (M.-G.K.); okwuosachibuzo3@kumoh.ac.kr (C.N.O.)

**Keywords:** YOLO, Dijkstra’s algorithm, defect detection, Nearest Neighbor algorithm, Genetic algorithm, laser displacement sensor

## Abstract

Product defect inspection is critical in industrial applications; however, it remains increasingly challenging in mass production environments, particularly for glossy or curved surface products. Conventional inspection of such surfaces typically relies on manual visual examination using gauges and operator judgment, which is time consuming and prone to inconsistency. This study proposes a robust defect detection framework for curved and reflective surfaces using a KEYENCE displacement laser sensor. The system integrates the Dijkstra algorithm, the Nearest Neighbor Algorithm, and a Genetic Algorithm to optimize the laser scanning path for structured image data generation. To validate the proposed framework, datasets were generated from both healthy and defective samples and used to train multiple deep learning models. A comparative analysis was conducted using YOLOv8, YOLOv9, YOLOv10, and YOLOv11 architectures. Experimental results demonstrate that YOLOv11 achieved the best overall performance, attaining an mAP50 score of 0.844 while also exhibiting lower computational complexity and faster inference.

## 1. Introduction

In modern industrial manufacturing, surface defect detection plays a critical role in ensuring product quality, reducing downtime, and improving production efficiency, particularly in high-precision sectors such as automotive, electronics, and metal processing [1]. Automated inspection systems are increasingly adopted to replace manual quality control due to their ability to provide higher inspection consistency and operational efficiency. Recent market analyses indicate that the global machine vision-based surface defect inspection market is experiencing rapid growth as manufacturers transition toward automated quality assurance systems [2]. However, the deployment of automated inspection technologies still faces several practical limitations, including high implementation costs, complex integration with existing production lines, and the requirement for specialized technical expertise.

One particularly challenging scenario involves the inspection of components with curved and highly reflective surfaces, such as polished metals or coated materials. In these environments, surface curvature and reflectivity often generate irregular lighting reflections, glare, and low image contrast, which significantly reduce the reliability of conventional vision-based inspection methods [3]. These optical challenges make the accurate detection of common industrial defects such as cracks, dents, and scratches considerably more difficult in real manufacturing environments. Furthermore, industrial automated inspection systems can cost approximately 150,000 to 500,000 USD per production line, which can limit their adoption, especially among small and medium-sized manufacturing enterprises [4]. Consequently, improving the efficiency and effectiveness of existing inspection systems has become an important research objective in industrial quality control.

**Figure 1:**
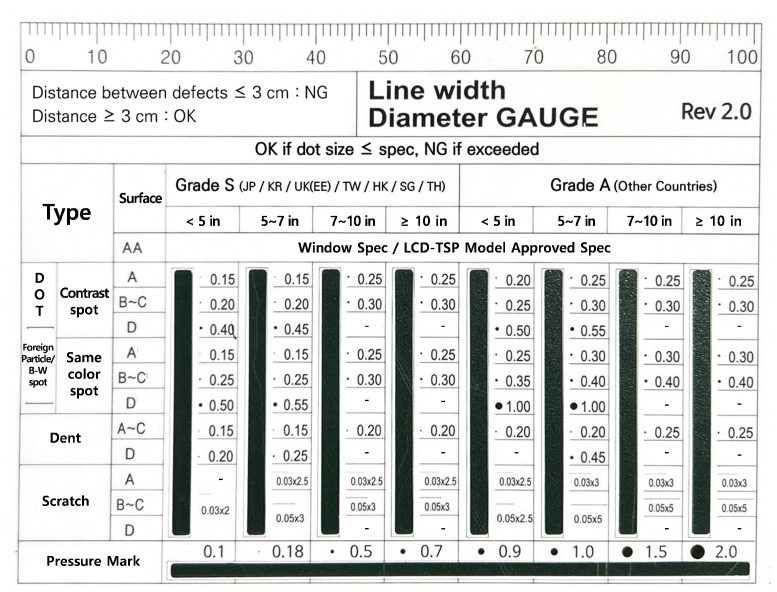
Line width diameter gauge.

Traditionally, operators have relied on visual and manual inspection methods to detect and classify faults on curved and reflective surfaces, where surface geometry and topology significantly influence defect visibility and detection accuracy [5]. A commonly used technique involves the use of a line width diameter gauge, as illustrated in Figure 1. In this method, operators visually compare the width and distance of detected defects to determine product quality. Because this process heavily depends on human judgment and operator experience, it introduces significant variability and reduces inspection reliability, particularly in high-volume manufacturing environments [6]. These limitations highlight the need for intelligent and automated inspection approaches capable of improving detection accuracy while maintaining efficient inspection processes.

Over time, continuous research efforts have led to the advancement of diverse defect detection techniques, encompassing optical and vision-based methods, precision laser and displacement sensing, and unique deep learning approaches powered by artificial intelligence (AI). Optical and vision-based methods, such as phase measuring deflectometry, structured light projection, polarization imaging, and photometric stereo, aim to optimize image acquisition and lighting conditions to accurately capture surface deviations and suppress glare [7]. These methods have been successfully employed in various studies. Cheng et al. [8] proposed a novel approach for defect detection on highly reflective surfaces using a phase deflectometry technique. Their study utilized polished metal plates, automobile door bowls, rear-view mirrors, and mobile phone glass cover plates as reference samples for evaluating the methodology. In their investigation, they introduced a gradient map representation based on the principle that surface gradients exhibit abrupt changes at defect locations on reflective surfaces. Similarly, Xiong et al. [7] combined the concepts of triangulation and infrared (IR) deflectometry to reconstruct the 3D topography of reflective surfaces without requiring complex integration operations. This resulted in a simple, fast, and cost-effective technique suitable for metal stamping inspection. Furthermore, Müller et al. [9] employed polarization filtering to minimize the effects of surface reflectivity. In their study, they captured two images with different polarization orientations and used the difference between them to isolate and suppress specular reflections on planar surfaces, thereby improving the accuracy of defect detection.

For defect detection on reflective surfaces using laser and displacement sensor techniques, methods such as confocal laser scanning, white light interferometry, and high-precision laser displacement sensing provide micrometer-to nanometer-level measurements for dimensional inspection and surface profiling, even on highly reflective materials [10,11]. The authors in [12] utilized confocal laser scanning and 3D reconstruction methods for defect detection on reflective surfaces. In their investigation, they applied a confocal laser scanning system, which achieved high-precision 3D imaging through the combination of galvo and piezo stage control for localized scanning and an XY stage for sub-aperture stitching across larger optical areas. Their experimental results on polished fused silica confirmed that their methodology can clearly visualize and quantify defect boundaries, positions, and cross-sectional dimensions, thereby providing a reliable foundation for quantitative assessment and optimization in optical manufacturing. In a more recent study, Yoon et al. [1] used a KEYENCE displacement sensor to generate data from a glossy, curved part of a product using a robot arm. The acquired data were subsequently used to train deep learning algorithms for defect detection, achieving the desired accuracy and reliability.

The implementation of advanced deep learning algorithms often depends on the nature of the dataset, which is heavily influenced by the technique used to generate the data [13]. However, some advanced deep learning algorithms perform better than others owing to their more sophisticated architectures. For instance, if the generated dataset contains images in which reflections completely obscure dented or scratched regions, defect detection becomes significantly more challenging for deep learning models. This occurs because reflective artifacts can mask critical visual features required for reliable defect identification. Furthermore, in situations where reflections are unavoidable, advanced deep learning models, such as generative networks and architectures incorporating attention mechanisms, have been developed to suppress reflective noise and emphasize regions where defects are more likely to occur, even when only partially visible [14,15]. However, these approaches do not always guarantee reliable detection performance due to the complexity of reflective surfaces. In many cases, deep learning models for defect classification on reflective surfaces require additional data preprocessing. Techniques such as data augmentation, image resizing, and orientation correction are commonly employed to improve data quality and enhance feature representation, thereby enabling the model to extract more discriminative features for accurate defect classification [1].

Similarly, curved surfaces have, over the years, proven to be challenging, as most data collection mechanisms are fixed and stationary, making it difficult to capture high-quality imagery of such surfaces. However, robotic arms with various degrees of freedom (DoF) have been utilized in studies, with six degrees of freedom representing the current standard in modern robotic systems [1,16,17]. These robotic arms ensure that the image sensor maintains an optimal orientation and distance relative to the curved surface, thereby capturing consistent views, minimizing reflections or occlusions caused by surface geometry, and improving defect visibility for downstream classification. Several studies have demonstrated the feasibility of 6-DoF robotic manipulators equipped with vision systems in industrial applications. For instance, Liu et al. [18] employed an eye-in-hand camera on a 6-DoF robot for part pose estimation; Wang et al. [19] developed an adaptive visual servoing framework for a 6-DoF robotic manipulator capable of tracking moving targets using a reinforcement learning-based PID controller; and a more recent study [16] utilized a 6-DoF robotic arm with RGB-D visual guidance for refueling tasks. Moreover, when dealing with curved and glossy surfaces, another study [1] combined a robotic arm with advanced imaging systems to enhance defect-detection quality.

Overall, for defect detection and classification based on image datasets, the application of diagnostic algorithms is essential for developing a robust and reliable inspection framework. Advanced image classification and detection approaches, including Convolutional Neural Networks (CNNs), Vision Transformers (ViTs), and Region-Based Convolutional Neural Networks (R-CNNs), have been widely used to extract discriminative features and improve recognition performance [20,21,22]. Deep learning architectures such as VGG16, ResNet, and EfficientNet have also demonstrated strong capability in feature representation and transfer learning for defect classification tasks [23]. For object localization and real-time defect detection, frameworks such as YOLO (You Only Look Once) and Faster R-CNN have achieved effective performance by balancing detection accuracy and computational efficiency in industrial inspection applications [24,25].

Despite these advances, reliable defect detection on curved and highly reflective surfaces remains challenging. Complex lighting reflections, irregular surface geometries, and low image contrast often degrade image quality and reduce the effectiveness of conventional inspection methods. To address these challenges, this study proposes an optimized inspection framework that integrates laser-based data acquisition with deep learning-based defect detection for curved and glossy surfaces. The proposed approach enhances defect visibility through structured laser scanning while enabling accurate and efficient detection using modern object detection architectures. Hence, the contributions of this study are listed thus:

- A comparative analysis of path planning algorithms to determine the most efficient approach in terms of computational time and traversal efficiency. This analysis aims to identify the most suitable algorithm for optimizing the scanning trajectory used during the data acquisition process.
- Development of an optimized hybrid path planning strategy that integrates the selected path algorithm with the Nearest Neighbor algorithm and a Genetic Algorithm to generate structured surface data using a laser displacement sensor mounted on a robotic arm. The proposed strategy enhances scanning efficiency by minimizing redundant movements while ensuring complete surface coverage.
- Evaluation of multiple YOLO-based single-stage object detection models for detecting dents and scratches using the dataset generated by the proposed scanning framework. YOLO-based architectures are employed due to their ability to achieve high detection accuracy while maintaining real-time processing capability.
- Comprehensive analysis of detection performance and computational efficiency to identify the most suitable model architecture for real-time defect inspection within the proposed framework.

## 2. Theoretical Background

### 2.1. You Only Look Once (YOLO)

YOLO is a state-of-the-art, single-stage object detection framework designed to perform real-time detection with high accuracy. Unlike traditional two-stage detectors that first generate region proposals and subsequently classify them, YOLO reformulates object detection as a single regression problem, directly mapping input images to bounding box coordinates and corresponding class probabilities in one forward pass through a convolutional neural network (CNN). This unified architecture significantly reduces computational complexity and latency, making YOLO highly suitable for real-time and embedded vision applications [26,27]. The YOLO algorithm operates by dividing the input image into a fixed grid of cells, where each cell is responsible for predicting a predefined number of bounding boxes along with confidence scores and class probabilities. Each bounding box prediction consists of spatial parameters describing the box location and size, as well as an objectness score that reflects the likelihood of an object being present within the box. The final detection results are obtained by combining these predictions and applying non-maximum suppression (NMS) to eliminate redundant and overlapping bounding boxes [26,27].

Though not as accurate as two-stage detectors, YOLO’s low latency combined with its competitive level of precision makes it more appropriate for real-time deployment. However, the accuracy limitations of early YOLO models have been significantly mitigated through successive architectural improvements. For instance, YOLOv1 demonstrated the feasibility of real-time object detection by achieving high inference speed with acceptable accuracy. Building upon this foundation, YOLOv2 enhanced detection performance by improving feature extraction through the adoption of the Darknet-19 backbone, the introduction of anchor boxes, and multi-scale training. YOLOv3 further improved detection accuracy by employing the deeper Darknet-53 backbone and incorporating feature pyramid networks, enabling more robust detection of objects across multiple scales [26,27].

Subsequent iterations continued this trend of balancing latency and accuracy. YOLOv4 [28] introduced extensive architectural and training optimizations, including cross-stage partial (CSP) connections, spatial pyramid pooling, and advanced data augmentation strategies, resulting in substantial performance gains without sacrificing real-time capability. Later versions, such as YOLOv5 through YOLOv8 [29,30,31,32], further refined the architecture by adopting anchor-free detection strategies, improved loss functions, enhanced feature aggregation, and multi-task learning support, enabling accurate object detection alongside tasks such as instance segmentation and pose estimation [27].

More recent variants, including YOLOv9 [33] and YOLOv10 [34], focus on improving training efficiency and inference robustness through architectural re-parameterization and optimized label assignment strategies. In particular, YOLOv10 adopts an NMS-free detection paradigm by enforcing one-to-one prediction matching during training, thereby eliminating the need for Non-Maximum Suppression at inference time and further reducing latency. Collectively, these advancements highlight YOLO’s continuous evolution toward achieving high detection accuracy while maintaining real-time performance. Building upon these architectural principles, ongoing and future YOLO developments are expected to incorporate further architectural refinements and efficiency-driven design strategies aimed at narrowing the accuracy gap between one-stage and two-stage detectors [29].

### 2.2. Dijkstra Algorithm

A foundational method in computer science for identifying the shortest paths in graphs featuring non-negative edge weights is Dijkstra’s algorithm, developed by Edsger W. Dijkstra. Celebrated for its computational efficiency and straightforward logic, this algorithm has become a cornerstone in graph theory [35,36,37]. Its utility extends to numerous real-world applications, including routing data across networks, calculating optimal routes in navigation systems like GPS, and solving complex optimization challenges. The algorithm operates on a greedy principle, making a series of locally optimal decisions at each stage, selecting the immediate shortest path, with the objective of arriving at a globally optimal solution for the entire graph [38,39]. The procedure starts by assigning a provisional distance value to every node. The source node is initialized with a distance of zero, acting as the starting point, while all other nodes are initially set to an effectively infinite distance, indicating they are unreachable. All nodes are initially marked as unvisited.

The core iterative process involves selecting the unvisited node with the smallest tentative distance and marking it as visited. For each neighboring node that is still unvisited, the algorithm calculates a new tentative distance by adding the current node’s distance to the weight of the connecting edge. If this newly calculated distance is lower than the neighbor’s existing provisional value, the shorter distance is recorded, and the current node is noted as the predecessor. This cycle repeats, systematically visiting nodes in order of increasing distance from the source, until all connected nodes have been visited. Upon termination, the algorithm has computed the shortest possible distance from the source to every other node in the graph.

Formally, within a given graph G=(V,E) with a vertex set *V* and an edge set *E*. Each edge connecting vertices *x* and *y* is assigned a non-negative weight, denoted w(x,y). In the execution of Dijkstra’s algorithm, two key arrays are maintained: dist[y] stores the shortest known distance from a source vertex *S* to vertex *y*, and pred[y] records the immediate predecessor of *y* on the current shortest path from *S*.

The algorithm commences with an initialization phase defined by Equation (1), which establishes the initial conditions for the search.

(1) Set:dist[S]=0,dist[y]=∞forally≠S

Here, *S* is the source node, and all other nodes *y* are initially at an infinite tentative distance. All vertices are initially marked as unvisited. The algorithm then proceeds iteratively. For the current node *x*, the algorithm examines each of its unvisited neighbors *y* and updates their tentative distances using the relaxation rule in Equation (2).

(2) dist[y]=min(dist[y],dist[x]+w(x,y))

Once all neighbors of *x* have been processed, it is marked as visited. The algorithm then selects the unvisited vertex with the smallest dist[·] value to become the next current node. This cycle continues until all vertices reachable from the source *S* have been visited.

### 2.3. Nearest Neighbor Algorithm

Nearest Neighbor Algorithm (NNA) is a fundamental heuristic approach in path optimization and combinatorial problems such as the Traveling Salesman Problem (TSP) [40]. NNA has been recognized for its simplicity and computational efficiency and has also become one of the most sought-after greedy algorithms in route planning and spatial optimization. Its applications extend across diverse fields, including logistics, autonomous navigation, manufacturing path scheduling, and network routing, where rapid estimation of near-optimal paths is essential [40,41].

The algorithm operates on a greedy selection principle, progressively constructing an optimized route through a series of locally optimal decisions. Starting from an initial node, NNA repeatedly selects the nearest unvisited node as the next step, under the assumption that minimizing immediate distance will yield a near-optimal global path [41,42]. This iterative process continues until all nodes have been visited, after which the path returns to the starting point, forming a complete cycle.

For each unvisited candidate point P[j], the Euclidean distance between the current point and P[j] is computed as:

(3) $$D\left(a,b\right)=\sqrt{{\left(x_{2}-x_{1}\right)}^{2}+{\left(y_{2}-y_{1}\right)}^{2}+{\left(z_{2}-z_{1}\right)}^{2}}$$

The point with the smallest distance is then selected, appended to the path, and marked as visited. This process repeats until all points are visited, and the total travel distance (TD) is obtained by summing the distances between consecutive points in the final route.

The strength of the Nearest Neighbor Algorithm lies in its computational efficiency and ease of implementation, making it particularly attractive for real-time or resource-constrained environments. However, its greedy nature can sometimes lead to suboptimal solutions, especially when early local decisions constrain global optimization. Despite this limitation, NNA remains a valuable baseline method and a practical foundation for advanced metaheuristic approaches such as Genetic Algorithms, Ant Colony Optimization, and Simulated Annealing. Its balance between simplicity and effectiveness continues to make it an enduring tool in modern path optimization research and applications.

### 2.4. Genetic Algorithm

The Genetic Algorithm (GA) is an evolutionary optimization technique inspired by the principles of natural selection and genetics. It operates as a population-based search method designed to identify near-optimal or globally optimal solutions within complex and nonlinear problem spaces [43,44]. In the context of coverage path planning, GA provides a robust approach for minimizing total travel distance and optimizing waypoint visitation sequences, particularly when the search space grows factorially with the number of waypoints [45,46].

GA begins by generating an initial population of candidate solutions, commonly represented as chromosomes that encode possible paths or waypoint sequences. Each chromosome is subsequently evaluated using a fitness function, which quantitatively assesses the quality of the solution based on predefined optimization criteria, such as total travel distance, path smoothness, or energy efficiency. Formally, the fitness value $F_{i}$ of an individual *i* can be expressed as:

(4) $$F_{i}=\frac{1}{D_{i}+\epsilon }$$

where $D_{i}$ denotes the total travel distance associated with the *i*th path, and ϵ is a small constant introduced to prevent division by zero. Higher fitness values correspond to shorter and more efficient paths.

Through an iterative evolutionary process, the algorithm applies three primary genetic operators: selection, crossover, and mutation. Selection identifies the fittest individuals based on their fitness scores, favoring those with better performance for reproduction. Crossover recombines genetic material from pairs of selected individuals to produce new offspring, promoting the exchange of beneficial traits. Mutation introduces small random changes in the offspring to maintain genetic diversity and prevent premature convergence to local optima [47]. This cycle of selection, crossover, and mutation continues across multiple generations, with each iteration gradually improving the overall population fitness. The process terminates when a convergence criterion, such as a maximum number of generations or a minimal improvement threshold, is met.

One of GA’s key advantages is its global optimization capability and adaptability to large, multidimensional, and nonlinear search spaces. Its parallel evaluation of multiple candidate solutions enhances convergence efficiency while reducing the likelihood of stagnation in suboptimal regions. Consequently, GA has become a preferred method for solving complex path planning, scheduling, and control optimization problems where traditional deterministic algorithms often fall short.

## 3. Proposed Framework Architecture

**Figure 2:**
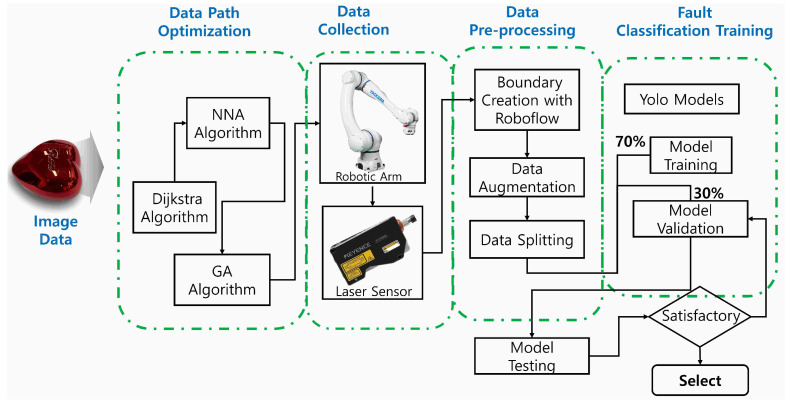
Proposed detection architecture.

The proposed fault classification framework consists of four major stages: data collection path optimization, data acquisition, data preprocessing, and model training and classification, as illustrated in Figure 2. Prior to the data acquisition stage, which involves mounting the KEYENCE LJ-X8200 displacement sensor (manufactured by Hangzhou Hikvision Digital Technology Co., Ltd., Hangzhou, China) on a YASKAWA robotic arm to overcome the limitations of stationary sensors when scanning curved surfaces, a path optimization stage is first implemented. This stage focuses on optimizing the robotic arm’s movement to determine the shortest and most efficient scanning trajectory using vertex extraction from a 3D CAD model. Among the evaluated path optimization techniques, the Dijkstra algorithm demonstrated the highest efficiency in determining the optimal scanning path. Furthermore, to reduce the computational complexity associated with the path optimization process, two additional algorithms, NNA and GA, were incorporated to further enhance computational efficiency.

### 3.1. Dijkstra Algorithm for Shortest Path Generation

Based on the 3D vertex coordinates of the given data, the shortest path from the starting point to the vertex farthest from it is extracted using Dijkstra’s algorithm, with the vertex list *P*, starting point coordinates start, and the total number of vertices *N* as inputs. To achieve this, Algorithm 1 was implemented to determine the farthest reachable vertex from a given start location while maintaining the fundamental principle of Dijkstra’s shortest-path search.

The algorithm begins by augmenting the set of destination candidate points $P=\left\{p_{1},p_{2},\ldots ,p_{N-1}\right\}\subset R^{3}$ with the starting coordinate to form a complete vertex list $P^{\prime }=\left[start\right]\cup P$ (step 1). Each vertex represents a potential 3D coordinate in the search space. Initialization follows, where all node distances are set to infinity, previous node indices are set to −1, and visitation flags are set to false, ensuring that no node is pre-assigned a prior path (step 2). The starting node’s distance is then initialized to zero, and it is inserted into a min-priority queue to serve as the initial reference for traversal (step 3). In the next stage, the Euclidean distances between all vertex pairs are computed to form a symmetric distance matrix *D*, where each element $D\left[i,j\right]={∥P\left[i\right]-P\left[j\right]∥}_{2}$ represents the spatial separation between two vertices (steps 4–6). Once the matrix is established, the main Dijkstra iteration loop is deployed. At each iteration, the node with the minimum tentative distance is extracted from the priority queue (step 7). If the node has already been visited, it is skipped to prevent redundant computations. Otherwise, the node is marked as visited, and the distances to all its unvisited neighbors are evaluated (steps 8–9). For each neighboring vertex *v*, an alternative path cost alt=d+D[u,v] is computed, where *d* is the current accumulated distance (step 10). If alt<dist[v], the tentative distance and predecessor index are updated, and the neighbor is re-enqueued for subsequent evaluation (steps 11–12).

This process repeats until the priority queue becomes empty, ensuring that the shortest-path distances from the starting vertex to every other vertex are fully determined (step 13). After completion of the shortest-path propagation, the algorithm identifies the vertex with the maximum shortest-path distance, representing the farthest reachable vertex under Dijkstra’s framework (step 14). This vertex index is denoted as $maxIdx=\arg\max_{i=1\ldots N-1}dist\left[i\right]$, and the corresponding accumulated distance is recorded as $D_{\max}=dist\left[maxIdx\right]$. The optimal route is then reconstructed by backtracking through the stored predecessor indices in the prev array, successively prepending each node to form the ordered trajectory $Route=[\pi_{0},\ldots ,\pi_{K}]$ (steps 15–17). The final outputs are thus the reconstructed path Route, which represents the sequence of vertices from the start point to the farthest vertex, and the associated maximum shortest-path distance $D_{\max}$. This algorithm effectively merges the classical Dijkstra search principle with farthest-point extraction logic, providing a computationally efficient and spatially optimal path, ideal for robotic scanning, geometric inspection, and precise 3D path planning tasks.
**Algorithm 1** Farthest-Distance Dijkstra Path Extraction.**INPUT:** $P=\left\{p_{1},\ldots ,p_{N-1}\right\}\subset R^{3}$ (destination candidates), start (start coordinates), *N* (number of points)**OUTPUT:** $Route=[\pi_{0},\ldots ,\pi_{K}]$ (start → farthest), $D_{max}$ (accumulated longest shortest distance)1:P←[start]∪P2:initialize ∀i:dist[i]←∞,prev[i]←−1,visited[i]←false3:dist[0]←0; Q← empty min-priority-queue; enqueue(Q,(0,0))4:**for** i=0 to N−1 **do**5:    **for** j=i+1 to N−1 **do**6:        $D\left[i,j\right]\leftarrow {∥P\left[i\right]-P\left[j\right]∥}_{2}$; D[j,i]←D[i,j]7:    **end for**8:**end for**9:**while** Q≠∅ **do**10:    (d,u)← extract-min(Q);11:    **if** visited[u] **then continue**12:    **end if**13:    visited[u]←true14:    **for** v=0 to N−1 **do**15:        **if** ¬visited[v] and D[u,v]≠0 **then**16:           alt←d+D[u,v]17:           **if** alt<dist[v] **then** dist[v]←alt; prev[v]←u; enqueue(Q,(alt,v))18:           **end if**19:        **end if**20:    **end for**21:**end while**22:$maxIdx\leftarrow \arg\max_{i=1..N-1}dist\left[i\right]$; $D_{max}\leftarrow dist\left[maxIdx\right]$23:Route←[]; u←maxIdx24:**while** u≠−1 **do** prepend *u* to Route; u←prev[u]25:**end while**26:**return** $Route,D_{max}$

### 3.2. Path Optimization Using a Hybrid Algorithm

To optimize the shortest path generation process, a hybrid approach combining NNA and GA was employed. For the 3D vertex set, Dijkstra’s algorithm was first applied to determine the shortest path from the starting point to each vertex. However, calculating paths to a single destination alone is insufficient to efficiently cover the entire search space. Therefore, a coverage path planning method based on the TSP was subsequently applied. The TSP seeks a path that visits all vertices exactly once while minimizing the total travel distance, but its high computational complexity makes finding the exact optimal solution practically infeasible. Consequently, the hybrid NNA–GA approach was used to efficiently approximate an optimal coverage path. Algorithm 2 presents the detailed implementation procedure of the proposed hybrid path planning optimization framework.
**Algorithm 2** Hybrid Nearest Neighbor–Genetic Algorithm (NNA-GA) for Path Optimization.**INPUT:**   *N* (population size), *G* (max generations), *T* (tournament size), *C* (crossover probability), μ (mutation probability)   $vertex\_list[\left(x_{1},y_{1},z_{1}\right)∼\left(x_{n-1},y_{n-1},z_{n-1}\right)]$, $start\_point(p_{0})$**OUTPUT:** $P_{best}$ (optimized path)1:vertex_list←start_point∪vertex_list2:n←|vertex_list|3:**for** i=0 to n−1 **do**4:    **for** j=0 to n−1 **do**5:        $D\left[i\right]\left[j\right]\leftarrow {∥vertex\_list\left[i\right]-vertex\_list\left[j\right]∥}_{2}$   ▹ Euclidean distance matrix6:    **end for**7:**end for**8:Compute initial NNA path: $P_{1}\leftarrow$ Nearest Neighbor path starting at start_point9:$PP_{1}\leftarrow \left[P_{1}\right]$              ▹ Add NNA path to initial population10:**for** i=2 to *N* **do**11:    Generate random permutation perm of [1...n−1]12:    $PP_{1}\leftarrow PP_{1}\cup \left[\left[0\right]+perm\right]$     ▹ Add N−1 random paths to population13:**end for**14:**for** g=1 to *G* **do**15:    **for** i=1 to *N* **do**16:        Fitness[i]←1/ total distance of $PP_{g}\left[i\right]$17:    **end for**18:    $P_{best}\leftarrow$ path in $PP_{g}$ with max fitness19:    $PP_{g+1}\leftarrow \left[P_{best}\right]$             ▹ Keep elite for next generation20:    **while** $|PP_{g+1}|<N$ **do**21:        Randomly select *T* candidates from $PP_{g}$ and choose best fitness as Parent122:        Randomly select *T* candidates from $PP_{g}$ and choose best fitness as Parent223:        Perform order crossover on Parent1 and Parent2 to create Child24:        Mutate Child with probability μ; keep start fixed25:        $PP_{g+1}\leftarrow PP_{g+1}\cup \left[Child\right]$26:    **end while**27:**end for**28:**return** $P_{best}$                      ▹ Optimized path

The algorithm begins by augmenting the vertex list of candidate points $vertex\_list=\{\left(x_{1},y_{1},z_{1}\right),\ldots ,\left(x_{n-1},y_{n-1},z_{n-1}\right)\}$ with the starting point $p_{0}$ to form a complete set of vertices $vertex\_list^{\prime }=\left[p_{0}\right]\cup vertex\_list$ (step 1). Each vertex represents a potential 3D coordinate in the search space. Subsequently, a symmetric Euclidean distance matrix *D* is computed for all vertex pairs, where each element $D\left[i,j\right]=∥vertex\_list^{\prime }\left[i\right]-vertex\_list^{\prime }{\left[j\right]∥}_{2}$ represents the spatial separation between two vertices (steps 2–4). The initial population for the genetic algorithm is generated by first computing a path using the NNA, starting from $p_{0}$, which constructs a heuristic path by sequentially visiting the nearest unvisited vertex (step 5). This NNA path is added as the first individual of the population $PP_{1}$, and the remaining N−1 individuals are generated as random permutations of the vertices while keeping the starting vertex fixed at the beginning (steps 6–7).

The main genetic algorithm loop is then executed over *G* generations to optimize the path length. At each generation, the fitness of each individual in the population is evaluated as the reciprocal of its total path distance, $Fitness\left[i\right]=\frac{1}{\sum_{k=0}^{n-2}D\left[PP_{g}\left[i\right]\left[k\right],PP_{g}\left[i\right]\left[k+1\right]\right]}$ (step 8). The individual with the highest fitness is selected as the elite path $P_{best}$ and directly propagated to the next generation to preserve the best solution found so far (step 9). The remainder of the next-generation population is filled using genetic operators. For each new individual, two parent paths are selected via tournament selection, where *T* candidates are randomly chosen, and the individual with the highest fitness is selected as a parent (steps 10–11). An order crossover is then applied to the two parents to produce a child path, preserving the relative ordering of vertices while ensuring that each vertex appears exactly once (step 12). The child path is subsequently mutated with probability μ by swapping two randomly selected vertices, keeping the starting point fixed at $p_{0}$ (step 13). This process repeats until the next-generation population reaches the desired size *N* (step 14).

After *G* generations, the algorithm outputs $P_{best}$, the path with the maximum fitness (minimum total distance), representing the optimized trajectory through all vertices starting from $p_{0}$ (step 15). By combining the heuristic advantage of NNA for initialization with the global search capability of the genetic algorithm, this hybrid approach efficiently identifies near-optimal paths for complex 3D path planning, inspection, and robotic navigation tasks. This hybridization allows the algorithm to leverage the quick, heuristic path provided by NNA while using the genetic algorithm to explore alternative paths that improve overall coverage efficiency, resulting in significantly shorter total paths compared to using either method alone.

### 3.3. Model Performance Evaluation Criteria

Evaluating a model is essential for establishing its robustness and generalizability. In this study, we assess the performance of our model using a comprehensive set of evaluation metrics. The global metrics employed include accuracy (Acc), precision (Pre), F1-score (F1), sensitivity (Sen), specificity (Spe), and mean average precision (mAP). These metrics are defined as follows:

(5) $$Ar=\frac{TP}{TP+FP+TN+FN}$$

(6) $$Ps=\frac{TP}{TP+FP}$$

(7) $$Fs=\frac{2*\mathrm{Sensitivity}*\mathrm{Precision}}{\mathrm{Precision}+\mathrm{sensitivity}}$$

(8) $$St=\frac{TP}{TP+FN}$$

(9) $$Sf=\frac{TN}{TN+FP}$$

(10) $$\mathrm{mAP}=\frac{1}{N}\sum_{x=1}^{N}AP_{x}$$

where FN, TN, FP, and TP represent the false negative, true negative, false positive, and true positive, respectively; while $AP_{x}$ and *N* represent the average precision of a given class *x* and the total number of classes, respectively.

Accuracy measures the overall classification performance of the model and is defined as the ratio of correctly predicted samples (both positive and negative) to the total number of predictions. Precision evaluates the reliability of positive predictions, indicating the proportion of correctly identified positive cases among all predicted positives. Sensitivity (also referred to as recall or true positive rate) quantifies the model’s ability to correctly identify actual positive instances, while specificity (true negative rate) measures its capability to accurately detect negative instances. The F1-score, on the other hand, represents the harmonic mean of precision and sensitivity, providing a single, balanced metric that accounts for both false positives and false negatives, making it particularly useful when dealing with imbalanced datasets. Collectively, these metrics offer a comprehensive evaluation of model performance under varying class distributions. Additionally, mAP, being a global performance indicator across all classes, computes the mean of the average precision values obtained for each class, where each $AP_{x}$ corresponds to the area under the precision-recall curve. A higher mAP value signifies better overall model performance, as it captures both precision and recall characteristics across multiple categories.

In this context, TP, FP, TN, and FN represent the fundamental components of classification performance. Specifically, TP (True Positives) denotes instances where the model accurately identifies a positive case, while TN (True Negatives) refers to correctly classified negative cases. Conversely, FP (False Positives) represents cases where the model incorrectly classifies a negative instance as positive, and FN (False Negatives) denotes cases where the model fails to identify an actual positive instance.

## 4. Experimental Setup and Framework Implementation

This section presents a detailed breakdown of the experimental setup, data description, procedure, model architecture, and visualizations, providing a comprehensive overview of the methodological framework adopted in this study.

### 4.1. Data Description and Path Optimization

**Figure 3:**
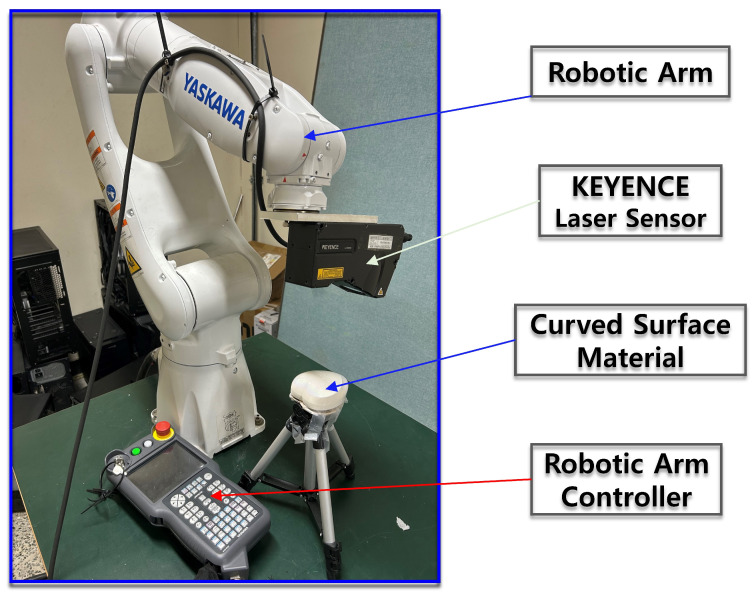
Experimental data collection setup.

The data collection process was conducted in our laboratory at Kumoh National Institute of Technology, Gumi, South Korea. The procedure comprised multiple stages aimed at establishing a robust framework to enhance model performance and efficiency. The dataset utilized in this study was obtained from a cosmetic manufacturing company located in Gumi-si, South Korea. The subject of investigation was a heart-shaped hairbrush case featuring curved and reflective surfaces, designed for aesthetic appeal and user elegance, as illustrated in Figure 3. The data collection process consisted of the following stages: extracting vertices from the 3D CAD model of the product, selecting a shortest-path search algorithm, optimizing the path using a hybrid approach, and feeding the optimized path coordinates to the laser sensor for data generation.

#### 4.1.1. Vertex Extraction from 3D CAD Conversion

**Figure 4:**
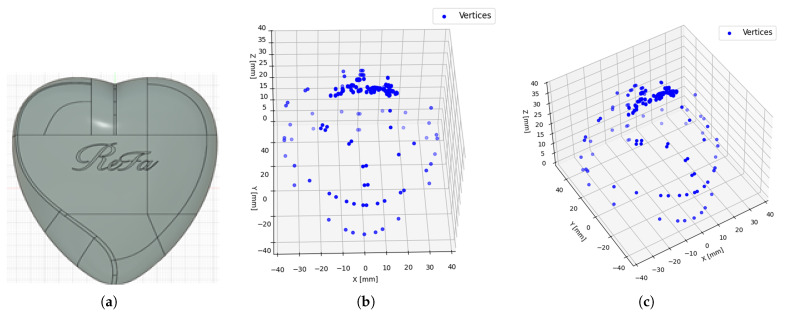
Overview of the 3D CAD file of the Heart-case (**a**) Full view, (**b**) Top view, (**c**) Side view.

To detect surface defects on the heart-case product, vertex coordinates were first extracted from its CAD model (STEP file), as shown in Figure 4. The CAD model was imported as a solid object; however, to simplify the analysis, only the exterior surface was considered, excluding internal volumes. Consequently, the extracted vertices correspond to the geometric intersections between edges and faces on the model’s exterior. These points serve as critical reference coordinates representing the product’s outer structure for subsequent defect detection. A total of 232 three-dimensional vertices were obtained from the heart-case STEP file, which were then discretized to support scanner positioning and define the robotic arm’s motion path. This preliminary stage establishes the spatial foundation upon which the path optimization process is built.

#### 4.1.2. Shortest Path Search Algorithm

After extracting the vertices from the CAD model, the next stage involved the comparative evaluation of multiple pathfinding algorithms to identify the most efficient and computationally effective method for shortest path determination. Among the algorithms considered, Dijkstra’s algorithm, Bellman–Ford, Floyd Warshall, and A* (A star) were implemented, analyzed, and summarized in Table 1. Each algorithm was evaluated based on key performance indicators, including time complexity, space complexity, and the ability to handle complex spatial configurations and varying node densities. The Floyd Warshall algorithm can compute the shortest distances between all vertex pairs; however, its time complexity of $O(V^{3})$ and high space requirements make it inefficient for the heart-case model containing 232 vertices. Similarly, the single-source Bellman–Ford algorithm exhibited lower computational efficiency under the same conditions due to its higher time complexity compared to Dijkstra’s algorithm.

Dijkstra’s algorithm, on the other hand, efficiently determines the shortest path from a single starting point to all reachable vertices and demonstrates superior performance in both time and space complexity, making it suitable for practical path generation. Although Dijkstra’s algorithm guarantees the shortest path by systematically expanding from the source node to all reachable nodes, it can incur higher computational overhead in dense graphs. In contrast, the A* algorithm introduces a heuristic function that combines the actual cost from the start node with an estimated cost to the goal, significantly enhancing computational efficiency while maintaining optimality under admissible heuristics.

**Figure 5:**
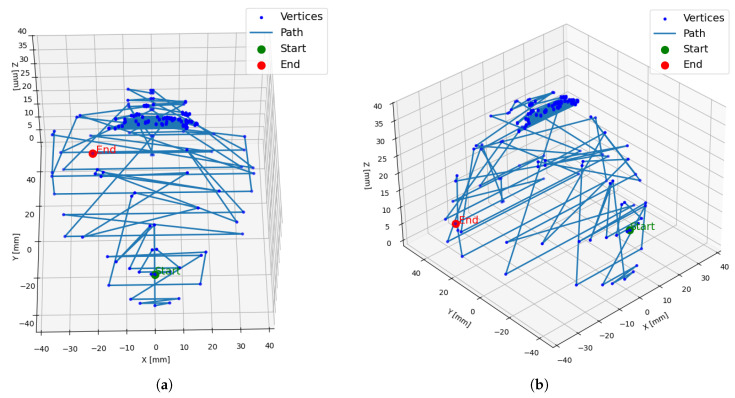
Overview of the Dijkstra’s algorithm generated path (**a**) Top view, (**b**) Side view.

Therefore, considering both efficiency and applicability, this study adopted Dijkstra’s algorithm as the shortest-path method for subsequent experiments. As described in Section 3.1, Dijkstra’s algorithm was implemented to analyze the 233 extracted vertices, including the starting point. The farthest vertex was identified as the 58th index (D_*max*_) with a cumulative distance of 2998.40 mm. An overview of the Dijkstra-based generated path is illustrated in Figure 5.

#### 4.1.3. Hybrid Path Optimization

**Figure 6:**
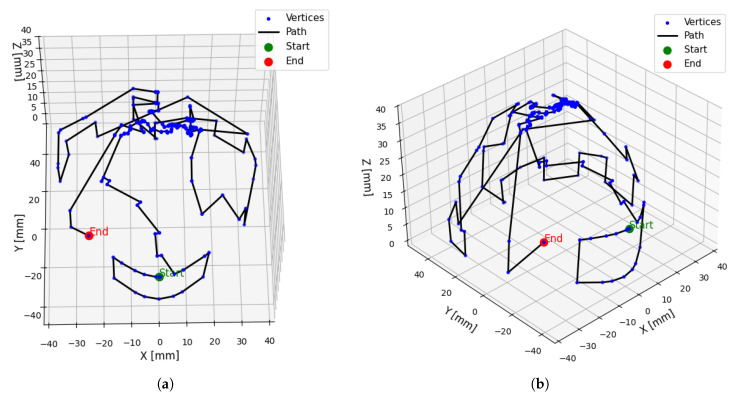
Overview of the Hybrid Generated Path (**a**) Top view, (**b**) Side view.

For the 3D vertex set, Dijkstra’s algorithm was first applied to determine the shortest path from the starting point to each vertex. Although effective for point-to-point navigation, this single-source approach was both computationally demanding and suboptimal for achieving complete spatial coverage in this instance. To overcome these limitations, a hybrid optimization strategy was developed, integrating a GA for global exploration with an NNA for local path refinement. This hybrid optimizer achieved a 73.2% reduction in total path distance, decreasing it from 2998.40 mm to 803.35 mm. The resulting optimized path, illustrating efficient traversal of the entire search space, is visualized in Figure 6.

### 4.2. Robotic Kinematic Modeling with Experimental and Simulation Evaluation

**Figure 7:**
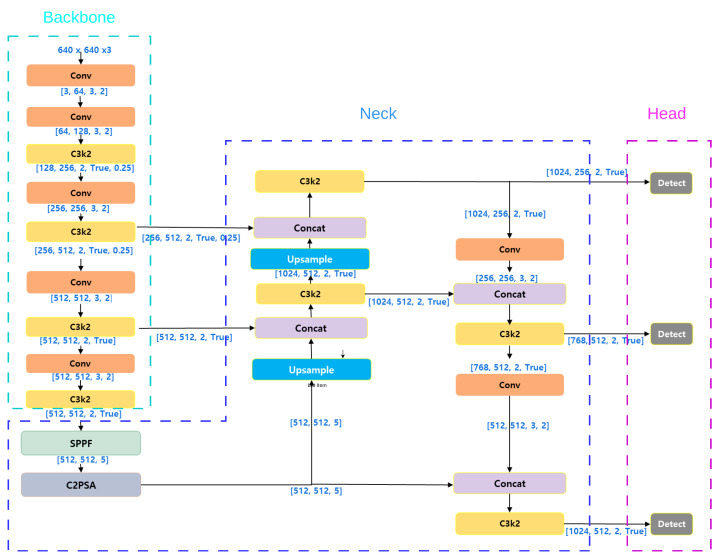
Architecture of the YOLOv11l object detection network [48].

The exterior of the heart case was modeled as a mesh-based surface, and measurements were conducted using a KEYENCE LJ-X8200 sensor, with the sensor specifications provided in Table 2. During the visualization process, strips were set with a width of 3.0 μm and a spacing of 3.0 μm to represent the spatial distribution around the path comprehensively. Additionally, due to the sensor’s characteristics, data acquisition requires satisfying the minimum measurement distance. Therefore, in the experiment, vertical measurement conditions were applied from the top of the heart case to calculate the end-effector coordinates of the robotic arm that meet the required distance. The experimental setup and the optimized path analyzed using MATLAB (R2025b) tools are shown in Figure 7. In both simulation and experiments, the end-effector joint link b of the Yaskawa 6-axis robot model was fixed such that its X-axis aligned with the global Z direction, ensuring that the laser scanner always faced downward. The origin of the heart-case was set at (0, 4, 2.4), and the starting coordinates of the end-effector were defined as (0.4, −0.05, 0.56). The sensor height was 95 mm, and since the original coordinate system was defined in meters, a scaling factor of 0.01 was applied for accurate conversion. The cumulative travel distance calculated from the experiment was 803.4 mm, closely matching the simulation result of 803.351 mm, demonstrating a high level of agreement between the experimental and simulation results.

### 4.3. Boundary Line Setup with Roboflow and Data Description

Roboflow was utilized for image annotation, dataset management, preprocessing, and export configuration. Defective regions were annotated using bounding boxes to accurately capture their location and spatial extent. This approach enabled precise representation of defect positions, which is essential for reliable region-of-interest definition and object localization.

The complete dataset consisted of 10,194 images, originally captured at a resolution of 2048×1024 pixels, and was divided into 8920 training images, 424 validation images, and 850 test images to ensure robust learning and unbiased performance evaluation. During preprocessing, automatic image orientation correction was applied, and all images were resized to 512×512 pixels using Roboflow’s (v3, 2025) export procedure, which scales width and height independently to standardize dimensions while preserving bounding box annotations. During YOLO model training, images were further resized to 640 × 640 pixels, which is the network’s input requirement. This two-step resizing introduces minimal geometric distortion and does not adversely affect defect detection performance.

Data augmentation was performed to enhance dataset diversity, generating three outputs per training example. Augmentation operations included horizontal and vertical flipping, hue variation within the range of $-13^{\circ }$ to $+13^{\circ }$, and exposure adjustment between −8% and +8%. All annotated data were exported in COCO (Common Objects in Context) format, selected for its native support of bounding box annotations and accurate spatial encoding.

### 4.4. Algorithm Parameters

We employed four variants of the YOLO object detection framework in this study, namely YOLOv8, YOLOv9, YOLOv10, and YOLOv11. The primary objective of this comparative evaluation was to determine the variant most compatible with the characteristics of our dataset, with the ultimate goal of developing an efficient, accurate, and robust detection model.

To ensure architectural fairness and comparable representational capacity, all models were implemented using their respective large-scale (L) configurations, YOLOv8l, YOLOv9l, YOLOv10l, and YOLOv11l, thereby maintaining similar parameter scales and computational complexity across all experiments. All implementations were conducted using the Ultralytics framework, a widely adopted open-source platform that provides standardized architectures, optimized training frameworks, and consistent evaluation protocols. Employing a unified framework ensured fairness in comparison by minimizing implementation bias and maintaining uniformity in data preprocessing, hyperparameter configuration, and training procedures across all variants. Consequently, performance differences observed among the models can be primarily attributed to architectural design choices rather than inconsistencies in implementation.

The architecture of the most recent variant evaluated in this study, YOLOv11, is illustrated in Figure 7. YOLOv11 advances beyond YOLOv10 primarily by enhancing representational strength rather than focusing solely on computational efficiency. Specifically, YOLOv11 incorporates deeper and more repeated C3k2 blocks, enabling stronger multi-stage feature refinement. At the deepest stage of the backbone, it integrates a Spatial Pyramid Pooling Fast (SPPF) module followed by C2PSA attention, thereby improving global context modeling and channel–spatial feature awareness. In contrast, YOLOv10 adopts a more efficiency-oriented design strategy.

## 5. Training and Evaluation

All models were trained for 100 epochs with a batch size of 8 on a workstation equipped with an NVIDIA GeForce RTX 3070 GPU (8 GB VRAM), an 11th-generation Intel Core i7-11700 processor (2.50 GHz), and 32 GB RAM, running a 64-bit operating system. The dataset description and the augmentation procedure applied after bounding box annotation using Roboflow are summarized in Section 4.3. The original dataset consists of 4247 images prior to augmentation, which increased to 10,194 images after augmentation. The Dent + Scratch class refers to instances where a product contains both dent and scratch defects simultaneously. Data augmentation was applied only to the training set to improve model generalization while preventing bias in the validation and test datasets. A summary of the dataset composition and augmentation process is presented in Table 3.

**Figure 8:**
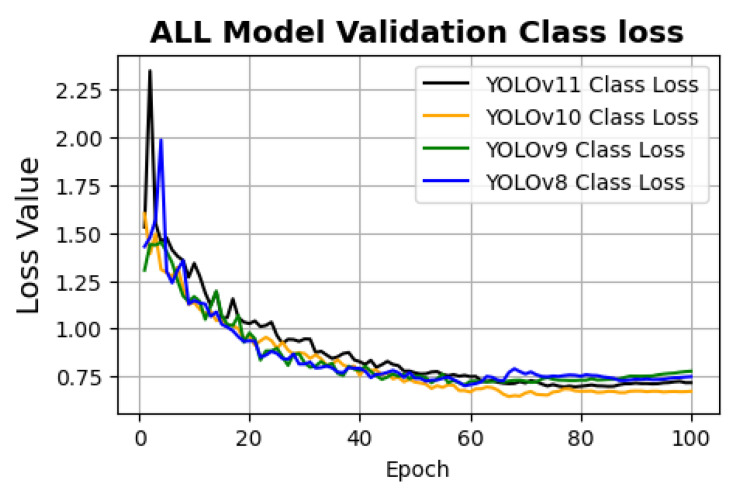
Validation classification loss curves for all evaluated models across training epochs.

Figure 8 presents the validation class loss curves of the four Yolo variants over 100 epochs. The class loss is presented to emphasize classification performance and inter-class discrimination. Since class loss directly reflects the model’s ability to correctly assign object categories, it provides a more relevant indicator for comparative analysis in this context.

The validation class loss curves indicate that YOLOv10 achieved the lowest validation class loss, demonstrating superior classification generalization compared to the other models. YOLOv11 followed closely with stable convergence behavior. YOLOv9 exhibited the highest validation class loss among all models. Although YOLOv8 and YOLOv9 showed lower class loss values during the early training epochs, this trend did not persist. In the later epochs, YOLOv10 and YOLOv11 displayed lower validation class loss, indicating improved generalization performance.

**Figure 9:**
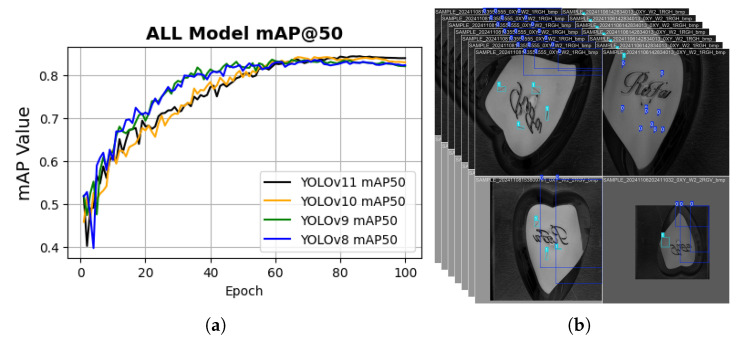
mAP50 assessment of all models. (**a**) Convergence trend; (**b**) Predicted samples.

**Figure 10:**
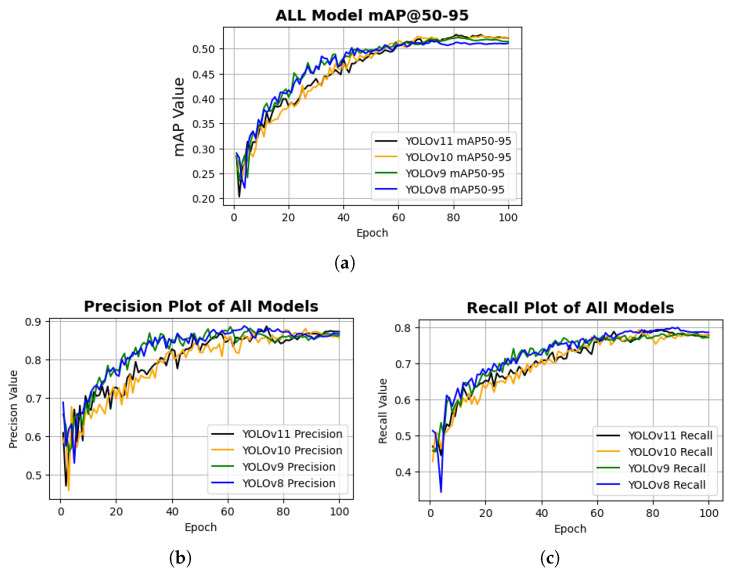
Training performance curves illustrating the learning behavior of the model during training: (**a**) mean Average Precision at IoU thresholds 0.50–0.95 (mAP50–95); (**b**) Precision; and (**c**) Recall. These curves demonstrate the convergence characteristics and detection performance of the model over training epochs.

To further assess the models, the training mAP50 was evaluated as a quantitative metric to measure detection accuracy and localization reliability. The comparative results are summarized in Figure 9. As observed from the plot, the convergence trend followed a trajectory similar to that of the class loss curves; however, in this case, YOLOv11 slightly outperformed YOLOv10, which contrasts with the validation class loss results. In addition, the training mAP@50–95, precision, and recall metrics are presented to provide a comprehensive evaluation of overall model performance and are presented in Figure 10.

From the visualization of mAP50–95 plots in Figure 10a, YOLOv11 and YOLOv10 achieved approximately similar performance, slightly outperforming YOLOv9 and YOLOv8. Notably, all models attained mAP50–95 scores above 0.50, which, given the challenges associated with glossy and curved surfaces, indicates strong localization performance. However, this trend was not observed in the precision and recall metrics. As shown in Figure 10b,c, all models exhibited nearly identical precision and recall values, suggesting comparable classification consistency across the evaluated variants.

**Figure 11:**
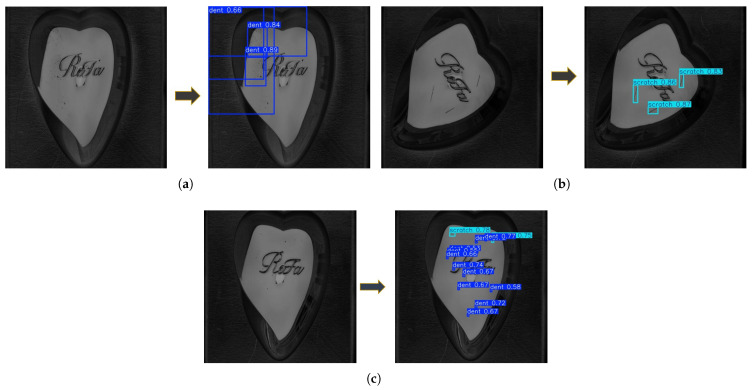
Visualization of defect detection results produced by YOLOv11 on the test dataset. For each example, the original image and the corresponding detection output are shown. (**a**) Dent defect example; (**b**) Scratch defect example; (**c**) Combined defect example. Bounding boxes indicate the detected defects.

A comprehensive evaluation of a detection model cannot be considered complete without assessing its performance on an unseen dataset. While training and validation metrics provide insight into convergence behavior and generalization trends, true model robustness is best determined through independent test inference. Therefore, all trained YOLO variants were evaluated on a separate test dataset to assess their real-world detection capability. A sample of the YOLOv11l detection performance is shown in Figure 11. The comparative performance metrics obtained during this inference stage are summarized in Table 4, providing a clear assessment of each model’s generalization capability, detection accuracy, and localization reliability under previously unseen conditions.

The results indicate that all evaluated models demonstrate strong generalization capability on unseen data, exhibiting relatively consistent performance across Precision, Recall, and mean Average Precision (mAP) metrics. Nevertheless, subtle yet meaningful differences emerge when detection accuracy and localization robustness are examined in greater detail.

Among the evaluated variants, YOLOv10 achieved the highest precision (0.883), indicating superior effectiveness in minimizing false positive detections. This behavior suggests a relatively conservative prediction strategy, prioritizing reliability in object confirmation. In contrast, YOLOv8 recorded the highest recall (0.794), reflecting an enhanced ability to detect a larger proportion of true objects, albeit with a slight compromise in precision.

With respect to detection performance at IoU = 0.5, YOLOv11 achieved the highest mAP50 (0.844), marginally outperforming the other variants. This result indicates a well-balanced trade-off between precision and recall, reflecting stable and reliable detection behavior under standard overlap criteria. Meanwhile, YOLOv9 demonstrated competitive performance with an mAP50 of 0.834, positioning it slightly behind the leading models.

Under stricter localization conditions (mAP50–95), all models exhibited nearly identical performance (0.525), suggesting comparable bounding-box regression capability across varying IoU thresholds. The observable gap between mAP50 and mAP50–95 indicates that while classification confidence is strong, precise localization at higher IoU thresholds remains a shared constraint across all evaluated architectures.

**Figure 12:**
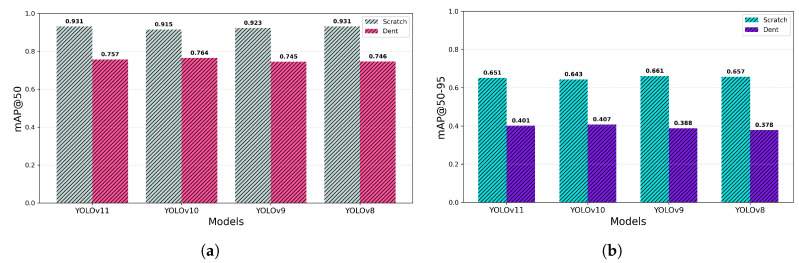
Class-wise evaluation performance of the model: (**a**) mean Average Precision at IoU = 0.50 (mAP50); (**b**) mean Average Precision across IoU thresholds 0.50–0.95 (mAP50–95).

In addition to the aggregated metrics, the class-wise performance analysis presented in Figure 12 provides further insight into model performance across defect categories (scratch and dent). For the scratch class, YOLOv11 and YOLOv8 achieved the highest mAP50 (0.931), indicating strong sensitivity to elongated surface defects. YOLOv9 showed slightly superior localization stability under stricter conditions (mAP50–95 = 0.661), suggesting marginally improved bounding-box refinement for scratch-type features. For the dent class, YOLOv10 achieved the highest mAP50 (0.764) and the highest mAP50–95 (0.407), demonstrating stronger detection and localization performance for compact deformation patterns. However, despite these category-specific advantages, its overall mAP50 remains slightly lower than that of YOLOv11. The class-wise analysis in Figure 12 also reveals that dent detection is inherently more challenging than scratch detection, as evidenced by consistently lower mAP50–95 values across all models. This suggests that compact defect geometries impose greater difficulty on bounding-box regression compared to elongated surface anomalies.

**Figure 13:**
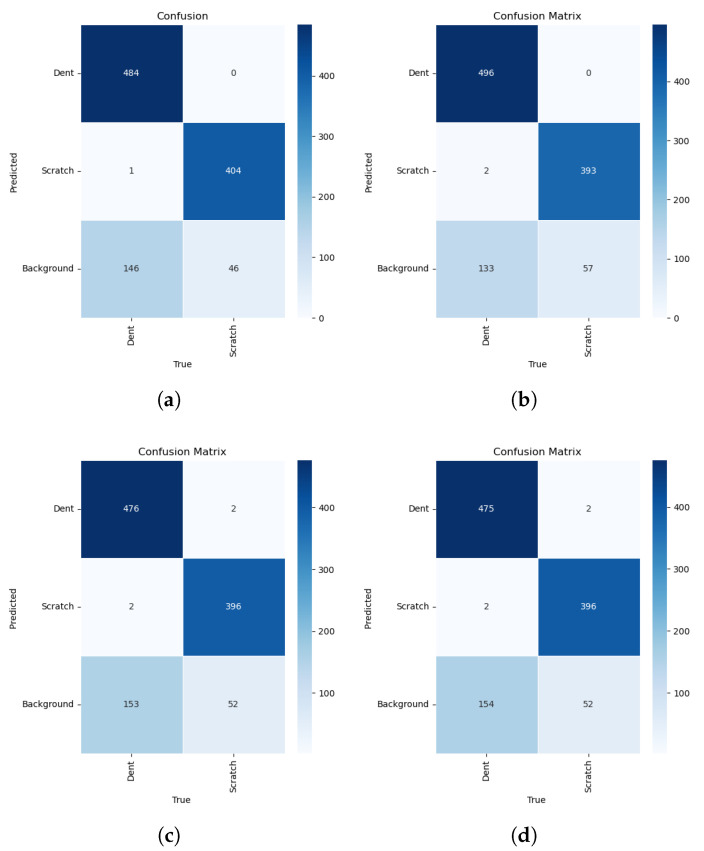
Confusion Matrix: (**a**) YOLO11l; (**b**) YOLO10l; (**c**);YOLO9l (**d**) YOLO8l.

The confusion matrices of the evaluated models are presented in Figure 13. The test dataset contains 1081 defect instances, including 631 dent samples and 450 scratch samples. These matrices illustrate the classification behavior of each YOLO variant by showing correct detections, interclass misclassifications, and missed detections. For YOLO11, the model correctly detects 484 dent instances and 404 scratch instances. Only one dent sample is misclassified as the scratch class, indicating strong discrimination between the two defect types. However, 146 dent samples and 46 scratch samples are predicted as background, suggesting that most errors arise from missed detections rather than class confusion. For YOLO10, the model achieves the highest dent detection with 496 correct instances, while scratch detection decreases slightly to 393 correct detections, with 57 missed samples. Although interclass misclassification remains minimal, the higher background predictions suggest reduced sensitivity to some scratch patterns. YOLO9 and YOLO8 show comparable performance, correctly detecting approximately 475–476 dent instances and 396 scratch instances, with only a few cross-class errors. However, both models exhibit higher numbers of missed detections, indicating lower detection sensitivity compared with the stronger variants.

Overall, all models demonstrate very low interclass misclassification, confirming that dent and scratch defects are well separated in the learned feature space. The main limitation across models is false negatives, where defects are predicted as background.

Furthermore, the computational assessment presented in Table 5 provides critical insight into the deployment feasibility of each model. While detection accuracy is essential, practical industrial implementation, particularly in real-time inspection systems, requires careful consideration of model complexity, floating-point operations (GFLOPs), and inference latency. Among the evaluated architectures, YOLOv11 demonstrates the most favorable efficiency profile. With 25.3 million parameters and the lowest computational burden (86.6 GFLOPs), it achieves the fastest inference time of 12.8 ms. This indicates superior architectural optimization, enabling high detection accuracy with reduced computational overhead.

Although YOLOv10 has the lowest parameter count (24.3 M), it requires significantly higher computational effort (120.0 GFLOPs), suggesting increased arithmetic intensity during forward propagation. This reduces its efficiency advantage despite its strong precision performance. YOLOv9 presents moderate computational demand (102.3 GFLOPs) with an inference time of 14.0 ms, positioning it between YOLOv11 and YOLOv10 in terms of efficiency. In contrast, YOLOv8 exhibits the highest model complexity, with 43.6 M parameters, 164.8 GFLOPs, and the slowest inference time (16 ms), indicating a substantially heavier computational footprint without proportional gains in detection performance.

When integrating computational efficiency with detection robustness (including class-wise evaluation), YOLOv11 maintains a clear overall advantage. Therefore, from a system-level perspective that considers both predictive performance and runtime efficiency, YOLOv11 achieves the optimal balance. This makes it the most suitable architecture for real-time deployment scenarios where computational resources and latency constraints are critical factors.

## 6. Discussion and Conclusions

This study presented an optimized data generation framework for laser displacement sensors through the integration of the Dijkstra shortest-path algorithm and a hybrid optimization strategy combining Neural Network Approximation and a Genetic Algorithm. The proposed approach was designed to address the challenges associated with curvilinear and glossy surface instances, where conventional scanning strategies frequently suffer from reflection-induced noise, path redundancy, and inconsistent sampling density. The Dijkstra-based formulation enabled global path optimality by modeling the scanning domain as a weighted graph, where the traversal costs were defined according to geometric curvature and surface reflectivity characteristics. This ensured efficient spatial coverage while minimizing redundant movements. To further enhance adaptability, the hybrid NNA–GA optimization mechanism was introduced to refine scanning parameters and sensor positioning. The neural approximation module provided rapid estimation of near-optimal configurations, while the genetic algorithm ensured global exploration and avoided premature convergence. Together, this integration improved convergence stability and robust solution for complex surface geometries.

Overall evaluation metrics demonstrated strong and consistent generalization in all models. YOLOv10 achieved the highest precision, indicating superior false-positive suppression. YOLOv8 obtained the highest recall, reflecting improved detection sensitivity. However, YOLOv11 achieved the highest mAP50, indicating the most balanced trade-off between precision and recall. Under stricter localization criteria (mAP50–95), performance across all models converged to similar values, suggesting comparable bounding-box regression capabilities. Class-wise evaluation revealed distinct performance patterns across defect categories. Scratch defects consistently achieved higher detection accuracy than dent defects across all architectures. For scratch detection, YOLOv11 and YOLOv8 achieved the highest mAP50, while YOLOv9 showed slightly stronger localization stability under stricter IoU thresholds. For dent detection, YOLOv10 demonstrated marginally superior performance in both mAP50 and mAP50–95. However, no single architecture consistently dominated across both defect types and evaluation thresholds.

Computational efficiency analysis further informed deployment suitability. YOLOv11 exhibited the lowest GFLOPs and fastest inference time while maintaining strong detection accuracy. In contrast, YOLOv8 incurred significantly higher parameter count and computational cost without proportional performance gains, whereas YOLOv9 and YOLOv10 showed intermediate efficiency. Considering detection robustness, class-wise stability (Figure 6), and computational complexity jointly, YOLOv11 provides the most favorable accuracy–efficiency trade-off, making it the most suitable model for real-time industrial deployment.

A limitation of the framework is the comparatively lower performance for dent defects, particularly under stricter IoU thresholds. Dent defects exhibit compact geometry, subtle deformation, and reduced edge saliency, making precise localization more challenging than for elongated scratches. Glossy surface reflections may further degrade bounding-box refinement. Future work may mitigate this limitation through targeted data augmentation, deformable convolutional mechanisms, depth-aware sensing integration, or attention-based feature enhancement. Future research will also focus on further validating the proposed defect detection framework on industrial molds with additional curved geometries beyond those investigated in the current study. This includes evaluating corresponding performance metrics once access to the full robotic arm and laser displacement sensing system becomes available. These extensions will provide a more comprehensive assessment of the adaptability and effectiveness of the framework in practical industrial scenarios, enhancing the persuasiveness of the results.

## Figures and Tables

**Table 1:** Comparison of shortest path algorithms.

Type	Objective	Source/Target	Time Complexity	Space Complexity
**Dijkstra**	Finds the shortest path from a single source to all vertices	Single source/all vertices	O((V+E)logV)	O(V+E)
**Bellman-Ford**	Supports negative weights for single-source shortest path search	Single source/all vertices	O(VE)	O(V+E)
**Floyd-Warshall**	Computes shortest paths between all pairs of vertices	All pairs/all vertices	$O(V^{3})$	$O(V^{2})$
A* Search	Heuristic-based search for optimal path to a destination	Single/multiple source–target pairs	O((V+E)logV)	O(V+E)

Note: *V* = number of vertices, *E* = number of edges.

**Table 2:** Laser displacement specification.

Laser Specification	
Product Name	LJX-8080
Measuring range Z-axis (height)	73 mm (±20.5 mm)
Measuring range X-axis (width)	35.0 mm
Repeatability Z-axis (height)	0.5 μm
Repeatability X-axis (width)	1.0 μm

**Table 3:** Dataset composition, split, and augmentation.

Dataset	Healthy	Dent	Scratch	Dent + Scratch	Images	After Augmentation
Original Dataset	1540	1011	1000	696	2973	–
Annotations	–	7878	5145	–	–	–
Train	–	–	–	–	2973	8920
Validation	–	–	–	–	424	424
Test	–	–	–	–	850	850
Total	–	–	–	–	4247	10,194

**Table 4:** Evaluation of models on test data.

Model	Precision	Recall	mAP50	mAP50-95
**YOLOv11**	0.854	0.789	0.844	0.526
**YOLOv10**	0.883	0.769	0.840	0.525
**YOLOv9**	0.872	0.778	0.834	0.525
**YOLOv8**	0.866	0.794	0.839	0.517

**Table 5:** Computation assessment.

Model	Parameter	GFLOPs	Inference Time (ms)
**YOLOv11**	25.3 M	86.6	12.8
**YOLOv10**	24.3 M	120.0	15.2
**YOLOv9**	25.3	102.3	14.0
**YOLOv8**	43.6	164.8	16

## Data Availability

The data presented in this study are available upon request from the corresponding author. The data are not publicly available due to laboratory regulations.
